# Fluctuation of suicide intent and other matters in psychosocial assessment post self-harm

**DOI:** 10.1192/bjb.2021.97

**Published:** 2021-12

**Authors:** Prasanna de Silva

**Affiliations:** Cumbria, Northumberland, Tyne and Wear NHS Trust, UK. Email: Prasanna.deSilva@cntw.nhs.uk

Professor Morgan's article rightly focuses on the fluctuations in suicidal intent among mentally ill people undergoing the various crises and vicissitudes of life. He emphasises the importance of repeated assessments, rather than relying on the initial one, to accommodate these fluctuations in intent.

He appears to have given up on prospects of predicting longer-term suicide risk but has not commented on the emerging body of evidence suggesting the effectiveness of combining an app-based questionnaire with inflammatory biomarkers such as interleukin subtypes, SAT1 and Toll-like receptor subtypes.^1^ These biomarkers probably reflect the degree of underlying stress which Professor Morgan describes, with some quantitative features provided in addition. These types of hybrid assessments should cover both the short- and longer-term risks but will not predict when (or under what circumstances) the lethal behaviour could take place. Consequently, mitigation needs simple strategies such as Dr Cole-King's suicide safety plan, a brief document co-produced with the patient, held by the patient and carer, describing what to do and who to contact if suicidal intent reaches a climax.^2^

Brief hybrid assessments might also be less intrusive and distressing to patients compared with the standard ‘psychosocial assessment’ carried out in emergency room settings, typically by junior psychiatric liaison staff and often under time pressure (including the 4 h wait and expectations of prompt bed clearance and discharge as the person is deemed ‘medically fit’). Often both the assessor and patient are aware that this is likely to be the only contact between them, further reducing the likelihood of frank disclosure of trauma and abuse; this is strongly associated with invalid assessments and completed suicide in the future.^3^

Patients also find repeated disclosures of personal details to multiple mental health staff frustrating and traumatic,^4^ along the lines of ‘why don't you look up the notes before speaking to me?’. Similar to the experience of repeated police interviews under implied caution (‘anything you say might be used for a future Mental Health Act assessment’), patients are (perhaps rightly) suspicious that the assessors are looking for discrepancies in the history to undermine the reliability of the person's account leading to suicidal thinking and/or self-harming behaviour, thereby making it easier to discharge (or dismiss) the patient seeking help.

Professor Morgan touches on in-patient (‘never event’) suicides,^5,6^ mainly involving patients who have either absconded or been given planned home leave, as major improvements to ward design (including shaving off door edges and securing windows, door handles and toilet equipment) have now taken place. He does not, however, suggest practical changes in ward policy, for example, the potential benefit of a face-to-face review within 24 h of being placed on home leave in order to check on basic needs (elegantly summarised by Maslow), as well as potential toxic relationships with close family members, who might be either over-controlling or otherwise pessimistic on the prospects of the patient moving from being a burden (a variation on therapeutic nihilism and malignant alienation, not often discussed in the literature).

Finally, the issue that I, as a clinician, struggle most with when debriefing assessors or looking at longer-term suicide mitigation is that suicide risk assessment is used primarily as a defensive tool by the assessor, possibly aided by the patient, who does not wish to upset the assessor or get him/her into trouble in the future. So, the ‘protective factors’ often highlighted in the assessment are documented without due diligence on how stable or permanent these are.

On occasion, a suicidal person will ‘blurt out’ a suicide plan he/she has been considering. Often, this communication is with a staff member of low rank, for example, a ward domestic or student nurse, simply based on their compassionate nature and their not being part of the ‘assessment brigade’. Typically, these patients will subsequently deny that they will carry out this plan, and at times they will deny ever having disclosed such a plan, but, given the circumstance or opportunity, they may use the plan. Alternatively, a person who has failed with a plan will deny wanting to repeat the action (for example, an overdose) but could use this as a learning experience to organise a variation or plan more violent methods such as jumping or hanging.

As Professor Morgan rightly states, an assessor needs to compassionately (and non-judgementally) ask whether alternative means have been considered following a failed suicide attempt. This is genuinely hard work and especially emotionally draining. Therefore, it is essential for staff assessing suicidal patients to be debriefed supportively and given sufficient time off (at least undertaking other duties) to regain their emotional composure.
